# Identifying novel candidates and configurations for human helminth vaccines

**DOI:** 10.1080/14760584.2021.1999810

**Published:** 2021-11-09

**Authors:** Rick M. Maizels

**Affiliations:** Wellcome Centre for Integrative Parasitology; Institute of Infection, Immunity and Inflammation, University of Glasgow, Glasgow, UK

**Keywords:** Cestode, nematode, trematode, extracellular vesicle, Th2 immunity

## Abstract

**Introduction:**

: Human infections with helminth worm parasites are extraordinarily prevalent across tropical and subtropical parts of the world, and control relies primarily on drugs that offer short-term suppression of infection. There is an urgent need for new vaccines that would confer long-lived immunity, protecting children in particular and minimizing community transmission.

**Areas covered:**

: This article discusses the development of helminth vaccines, from the first successful veterinary vaccines that demonstrated the feasibility of inducing protective immunity to helminths, to more recent initiatives to test human helminth antigens. The field has focussed primarily on evaluating individual antigens that could constitute targets amenable to antibody attack to inhibit parasite establishment. In a new direction, vaccines employing extracellular vesicles released by helminths have also given exciting results.

**Expert opinion:**

: Taking into account the complex life cycles and sophisticated immune evasion strategies of many helminths, a combination of antigens and approaches designed to target essential functional pathways of the parasite will be required to achieve a high level of protection in future anti-helminth vaccines.

## 1. Introduction

Vaccines against human helminth parasites remain one of the greatest challenges in global medicine [1,2]. Helminth roundworm (nematodes) and flatworm (cestodes and trematodes) infect over a billion people across many low/middle-income countries [3], with high prevalence in children and a myriad of pathological effects [4]. Drug treatments are of limited efficacy, with populations rapidly reinfected and showing little sign of acquired immunity from natural exposure to infections [5,6]. Hence, there is a burning need for vaccines that would interrupt transmission and confer lasting benefits on many of the poorest communities in the world [7,8].

Efforts to generate vaccines against helminths date back 50 years to the development of a dog hookworm vaccine using irradiated infective larvae of *Ancylostoma caninum* [9,10]. While not a commercial success (as the vaccine did not entirely prevent transmission), this demonstrated that vaccine-induced immunity was possible, and paved the way for a similar irradiated larval vaccine (Huskvac) for lungworm (*Dictyocaulus viviparus*) in cattle [11]. A separate initiative, also in the veterinary arena, led to the development of a vaccine based on purified intestinal antigens of *Haemonchus contortus* (the ‘Barber’s Pole’ worm), now marketed as Barbervax [12,13]. Finally, successful recombinant vaccines have been pioneered against cestode tapeworms of livestock [14], now marketed in a number of tropical countries as CysVax (for *Taenia solium* in pigs) and Hidatil (for *Echinococcus granulosus* in ruminants).

Despite these successes, we still lack vaccines for human helminth infections. In part, irradiated larval strategies could not be applied to humans on any scale, and there are far greater regulatory hurdles than in the veterinary setting. Nevertheless, hundreds of candidate antigens have been tested in model systems for filariasis [15,16], schistosomiasis [17–19], soil-transmitted nematodes [20] and other human helminth parasites [21], with trials in endemic populations under way for hookworm and schistosomiasis [22]. But there remain some fundamental obstacles to achieve fully effective vaccines against human helminth parasites.

## 2. Challenges for helminth vaccines

A primary consideration is the nature of helminth infections, in which parasites migrate through different tissues, maturing from infective stages to adult worms, presenting a ‘moving target’ in more senses than one. Secondly, helminths are generally large, resilient organisms that may require a sustained and multi-pronged immune assault, rather than the one-off ‘lethal hit’ that the immune system can deliver to a virus-infected cell. Thirdly, they express multiple immune evasion strategies, at many levels and most likely in a redundant fashion, that subvert and defuse vaccine-induced immunity.

These factors militate against the conventional single subunit vaccine development approach that has proved successful for microbial pathogens. In contrast, for a single helminth antigen to induce effective protective immunity, that target would have to be sufficiently expressed over time, and be essential for survival if a vaccine were not simply to select for parasites that lose expression of the antigen. It is also to be expected that many helminth antigens will show polymorphisms across natural populations; while this dimension is rarely evaluated, a combination approach will minimize the danger of vaccine failure due to antigenic variation of the key target epitope.

A further problem with single subunit strategies is that all induce partial rather than complete immunity. Even in animal models, with little variation in host or parasite, immunization with individual antigens is often considered successful if 50% worm load reductions are achieved, and trials in endemic human populations with single antigen vaccines have so far been disappointing [23]. Vaccines that reduce worm loads by 50% may not compromise the ability of helminths to down-regulate the immune system, and will do little to break transmission; those that block egg production while leaving adult worms unscathed may do little to benefit the individual receiving the vaccine. Hence, a synergistic approach will often be needed. The cestode vaccines that succeed with a single target (To45 or Eg95), are exceptional as the target oncosphere stage is small enough (~20 µm) to be lysed by complement, and expresses a minimal number of products essential for its invasion of the intestinal mucosa [24]. Among other helminth organisms, the schistosome protein Sm-p80 (calpain) individually induces high levels of protection in baboons and is currently awaiting clinical trials [25,26], but no other single protein has proved so effective. Most anti-helminth vaccines may therefore need to be multi-component ‘cocktails,’ as successfully applied to a sheep nematode [27] and more recently explored with the human filarial parasite *Brugia malayi* [28]. Although posing considerable obstacles, for example, if each individual component must be produced and validated under GMP conditions, new technologies such as mRNA vaccines [29] offer more rapid development and could facilitate the combination of multiple antigens into a single immunogen.

A widely discussed challenge for helminth vaccines is the immunization of individuals who have been previously exposed, and/or are currently infected with parasites that profoundly manipulate the host immune system [30]. Preexisting IgE responses in adult recipients of the *N. americanus* ASP-2 antigen led its discontinuation in the first iteration of the human hookworm vaccine [31], although it may still be suitable for vaccination of infants prior to their first exposure. Developing successful vaccines against helminths also requires appreciation of the immunomodulatory effects that may persist in dampening immunity, particularly as infections are known to inhibit responses to microbial vaccines [32,33]. Thus, prior infection could confound vaccination either by hyper-reactivity to vaccine antigens, or conversely by ‘immunoregulatory memory’ that would represent a form of antigen-specific immune tolerance that could prevent responses to a helminth vaccine.

## 3. Mechanism-led approaches

Historically, helminth antigen identification strategies have been empiricalfor example, screening for antigens recognized by serum antibodies from exposed but uninfected (‘putatively immune’) individuals, with less consideration given to biological context (e.g. expression through the life cycle, essential function and accessibility to the immune system. With the explosion in helminth genomics, transcriptomics and proteomics, candidate antigens are plentiful and require thoughtful criteria to select the most effective.

This brings to the fore the question of mechanism; a clear understanding of how the immune system targets and eliminates helminth parasites is required for a rational choice of vaccine antigens. Again, adopting paradigms of antibody-mediated cytotoxicity or lytic T cells from the microbial world can be misleading – rather than sudden death, helminths are degraded more slowly and by the mass effect of innate immune cells, either by swarming and surrounding in the tissues, or bathing in mucus and inhibitory products in the lumen of the gut. Over time, these progressively compromise parasite fitness, first reducing egg production (the ‘anti-fecundity’ effect) and eventually causing the death of the whole organism [34]. Choosing antigens that represent key vulnerabilities of the parasite, as considered below, is of central importance; so too is the recognition of responder T cell modes (primarily but not always Th2). However, few discussions of helminth vaccines have as yet included pathways to promote mobilization of the innate effector populations (macrophages, eosinophils, and even neutrophils) that would be required to block worm establishment [35].

Across the helminth field, two lines of approach have been able to integrate mechanism with rational antigen selection. A long-standing example is that of targetting intestinal enzymes of hematophagous parasites such as *H. contortus* in sheep, and *N. americanus* in humans, or of flatworms established in the vasculature or tissues [36,37]. Here, the logic is that internal parasite constituents exposed on their intestinal membrane represent ‘hidden antigens’ which have not been subject to immune pressure, and for which even infected individuals have no preexisting antibody. Hence, vaccination will generate antibodies that, when ingested with blood by the parasites, will interfere with worm nutrition and eventually cause their demise. This principle underlies the Barbervax vaccine, using industrial-scale extraction from native parasites, and the second generation human hookworm vaccine currently under evaluation [38]. The latter is a bi-valent entity with recombinant APR-1 (aspartyl protease which digests hemoglobin) and GST-1 (gluathione *S*-transferase which detoxifies heme). In combination, antibodies are thought to prevent parasites from digesting blood normally. While this is an attractive strategy, with proven efficacy in the case of *H. contortus*, it is limited to those species that feed on blood, and targets the mature stages of those species, rather than immature, tissue-migratory larvae. In addition, if the target antigens are never presented to the immune system, there will be no natural boosting by subsequent parasite infection or exposure, necessitating re-vaccination to maintain antibody levels.

Further examples of this physiology-led approach are found in the current generation of Schistosome vaccines, including Sm-p80, which is highly expressed on the adult worm tegument being implicated in surface turnover and immune evasion, and as mentioned above generated a high level of protection in baboons [25,26]. Other candidates being actively pursued are tetraspanin (TSP)-2, again linked to the tegumental membrane, FABP/Sm-14, a widely expressed fatty acid binding and uptake protein, as well as the glutathione *S*-transferase Sh28GST, which in Schistosomes is also involved with muscle function [30]. Interestingly, there are suggestions that the existing *S. mansoni* vaccine antigens should be employed in combination [22].

A second mechanistic approach with a strong immunological foundation has been to target the suite of factors that helminths release to modify their environment and down-modulate the host immune system. Indeed, many of the first experimental vaccines in different helminth models utilized released molecules (termed excretory/secretory or ES products) and achieved high levels of protection [39]. However, these are generally complex mixtures and in very few cases have individual ES proteins been shown to induce protective immunity. Most probably, the redundancy of parasite products is responsible for lack of efficacy, returning the discussion to the need for multicomponent vaccines. In a study on a murine model system, *Heligmosomoides polygyrus*, immunization with ES products elicited sterile immunity to challenge infection; from the mixture, a combination of 3 proteins (VAL-1/2/3) were sufficient to drive immunity [40]. Interestingly these are members of the same gene family as *N. americanus* ASP-2, which was an earlier human vaccine candidate, and a number of homologs tested as vaccines for veterinary nematodes [41]. It remains to be determined if there is functional homology between members of this gene family in the different host-parasite combinations, but their prominence as effective immunogens does indicate a key role in parasite modulation of the host [41].

## 4. Extracellular vesicles – a new target

Most recently, a new dimension in helminth vaccines has emerged with the discovery that many parasites release within their ES material not only soluble macromolecules, but lipid-bound vesicles containing a cargo of proteins and small RNAs [42–44]. Importantly, these extracellular vesicles (EVs) exert significant immunomodulatory effectsfor example, inhibiting the expression of the receptor for IL-33 that is required to initiate the type 2 immune response [43], and down-regulating the activation of macrophages involved in innate immunity to helminths [45].

In the model *H. polygyrus* system, secreted EVs were found to evoke a strong serum antibody response, and a high degree (>80%) of protective immunity when administered with alum adjuvant [45]. *In vitro*, anti-EV antibodies promoted uptake of vesicles by macrophages into the lysosomal pathway, disabling them from exerting modulatory effects. Thus, in the presence of antibodies macrophages were protected from immunosuppression and retained their type 2 profile. Subsequently, further studies reported that immunization with EVs from the trematode *Opisthorchis viverrini*, and the nematode *Trichuris muris*, induce a protective immune response [46,47].

Analysis of the proteins associated with *H. polygyrus* EVs revealed a prominent set of components associated with the apical intestinal epithelium of nematodes [43], including homologs of antigens represented in Barbervax, the preparation of *H. contortus* gut material, such as H11, as well tetraspanins discussed above which are already candidate vaccines for *S. mansoni*. Immunogenic tetraspanins also were abundant in a proteomic analysis of *S. haematobium* EVs, alongside other vaccine candidates such as GST [48], giving credence to the possibility that immunization with these antigens targets EVs as well as parasite tissues.

Furthermore, vaccination with individual EV antigens, rather than intact EVs, may suffice to generate an antibody response that will neutralize the total immunomodulatory cargo – including small RNAs [44]. In this setting, parasites release combinations of immune modulators that are packaged into the EVs, to target host cells such as the macrophage and block protective immunity. Due to the discrete nature of the vesicle, antibody binding to any of its surface epitopes should result in uptake of EVs by phagocytes, and thereby destruction of the whole entity. Hence, targetting EVs by vaccinating against a single surface antigen could achieve exactly the combinatorial effect that is required for immunity. In practice, an anti-EV vaccine would be most potent targetting multiple-surface constituents, especially if the vesicles are heterogeneous with respect to membrane protein expression, or (as it likely) differ in composition between immature and mature parasite stages.

## 5. Expert opinion

Historically, the field of human helminth vaccine development has been dominated by the search for individual antigens that would generate protective immunity against a single species of parasite. However, helminths are complex organisms with sophisticated immune evasion mechanisms, and immunity is likely to require a co-ordinated attack on many fronts. Hence, we may already have in hand key antigenic targets but need to consider how to employ them to best effect, in combinations and with optimal adjuvants that induce the most effective mode of immune response. Hence, it may be that the era of discovering new candidate antigens for the major human helminths will soon draw to a close, replaced by a strategy of selecting, combining, and configuring the known candidates, potentially through modifications to maximize immunogenicity and minimize any safety concerns.

Whether based on conventional protein antigens, or EV-based components, vaccines can be developed that target both larvae and adults, preempting both pathology and transmission; immunity to helminths can act to degrade their fitness, as observed in ‘anti-fecundity immunity’ in which surviving adult worms produce fewer eggs. Accordingly, it may be expected that even if parasites survive immune attack as larvae, they will be less able to resist expulsion once they mature to adults. In this setting, multi-stage vaccines should show a synergistic effect, and would be particularly desirable where tissue-migrating larvae are as pathogenic as intestinal adults of the same species.

In the case of functional ‘hidden antigens’ and those from EVs, there is more likelihood of conserved epitopes shared across species so that a pan-helminth vaccine might be possible, and there is a further possibility of a compound vaccine presenting homologous antigens from multiple species to create the pan-helminth vaccine discussed previously [49]. Strategically, a vaccine that targetted multiple helminth species would be not only of logistical value in delivering protection to low-income communities, but would also avoid the possibility that clearance of one helminth species could provide greater opportunities for others.

Combinatorial vaccines, however, imply a substantial growth in permutations that will require testing, shining a greater spotlight on screening and trial capacity. The problem is accentuated because animal models are distant from the human setting for most helminths, although *Schistosoma mansoni* infects rodents and the human and mouse *Trichuris* species are closely related. On a positive note, veterinary helminth vaccines are being actively pursued [50] and can act as excellent pathfinders across a range of issues, from antigen validation, selection of combinations, and optimal mode of immune response induction, each against a backdrop of a pan-helminth vaccine agenda. Moreover, exciting progress has been made on controlled human infection with hookworm [51,52,53] and schistosomes [54] that will more quickly and rigorously evaluate front-running vaccine formulations.

Undoubtedly, the pipeline to realizing a final vaccine for human use is long and difficult, with multiple hurdles to progress each candidate through the ‘critical path’ to approval [2]. Possibly, a half-way house can be established in which first-generation vaccines that do not yet reach full efficacy can be combined with existing drug administration regimens to dramatically lower the burden of human helminth infections. Clearly, this issue is becoming ever more urgent to address, and only sufficient mobilization of resources may stand between us and final realization of new vaccines against these parasitic scourges.

## Article highlights

- Helminth vaccines have been shown to be effective in the veterinary arena
- Subunit vaccines with single antigens are less likely to succeed for human helminth parasites
- Vaccine design should be informed by mechanistic understanding of immunity
- Combinatorial antigens are likely to be required for effective vaccination, and would offer advantages of vaccines targeting multiple stages and species
- Extracellular vesicles may represent conserved targets suitable for vaccine development.
